# Excessive Gaming and Online Energy-Drink Marketing Exposure Associated with Energy-Drink Consumption among Adolescents

**DOI:** 10.3390/ijerph191710661

**Published:** 2022-08-26

**Authors:** Chung-Ying Yang, Fong-Ching Chang, Ru Rutherford, Wen-Yu Chen, Chiung-Hui Chiu, Ping-Hung Chen, Jeng-Tung Chiang, Nae-Fang Miao, Hung-Yi Chuang, Chie-Chien Tseng

**Affiliations:** 1 Tri-Service General Hospital, Taipei 11490, Taiwan; 2 Department of Health Promotion and Health Education, National Taiwan Normal University, Taipei 10610, Taiwan; 3 Graduate Institute of Information and Computer Education, National Taiwan Normal University, Taipei 10610, Taiwan; 4 The Graduate Institute of Mass Communication, National Taiwan Normal University, Taipei 10610, Taiwan; 5 Department of Statistics, National Chengchi University, Taipei 11605, Taiwan; 6 School of Nursing, Taipei Medical University, Taipei 11031, Taiwan; 7 Department of Public Health, Kaohsiung Medical University, Kaohsiung 80708, Taiwan

**Keywords:** energy drinks, excessive gaming, online marketing exposure, literacy

## Abstract

In this study, we examined excessive online gaming by adolescents and the resultant effects of their exposure to the online marketing of energy drinks and alcohol, and whether marketing literacy could serve as a mitigating factor. This cross-sectional study was conducted in 2020. Data were obtained from a sample of 2613 seventh-grade students from 30 middle schools in Taiwan. A self-administered questionnaire was conducted. The results showed that nearly 18% of the adolescent respondents had used energy drinks, while 75% reported seeing energy-drink advertisements on the internet in the past year. Multiple regression results indicated that factors such as being male, reporting excessive gaming, being exposed to higher levels of online energy-drink marketing, and reporting alcohol use were positively associated with energy-drink consumption. A higher level of online energy-drink marketing-affective literacy, however, was negatively associated with energy-drink consumption. In conclusion, factors that predicted energy-drink consumption among adolescents included excessive gaming and exposure to online energy-drink marketing, but marketing-affective literacy tended to lessen the impact of such advertising.

## 1. Introduction

Energy-drink manufacturers’ marketing strategies that target youth groups have been effective, and the number of adolescents who use energy drinks is increasing [1,2]. Consumption of energy drinks among adolescents has grown substantially in several countries such as the United States [3], Finland [4], and Norway [5]. A European survey shows that adolescents consume an average of 2 L of energy drinks per month, which leads (68%) all other age groups [6]. A Canadian study found that 62% of adolescents had used energy drinks in the past year [7]. Few studies have investigated the use of energy drinks among Taiwanese adolescents, while one study showed that half of college students had used energy drinks in Taiwan [8]. Energy drinks frequently contain high levels of caffeine, and many ingredients that have not been sufficiently studied and are not regulated could have potentially serious adverse effects on children and adolescents [9,10]. Energy-drink consumption has been linked to caffeine intoxication [11], obesity [12], dental erosion [13,14], headaches [15], stomach aches, insomnia [16], cardiovascular disease [17], and allergic diseases [18]. The use of energy drinks has also been associated with mental health problems [19] such as depression [7], anxiety symptoms, hyperactivity/inattention symptoms [20], and suicide ideation [21].

Unhealthy food and beverage marketing, such as that for energy drinks, is prevalent on the Internet [22,23]. An Australian study has shown that energy drinks are popular on digital platforms [24], while a UK study showed that the use of energy drinks by adolescents could generally be linked to social activities, sports, and computer gaming [25]. Due to the rapid development of digital marketing, energy-drink manufacturers mainly use the Internet for promotion via social media platforms or online games [23,24,25,26]. In Canada, about half of adolescents have seen energy-drink advertisements on the Internet, where energy-drink advertisements are perceived as targeting youth [27]. Exposure to energy-drink marketing is common among Canadian youth and significantly more prevalent than exposure to educational messages [28]. Research has associated frequent energy-drink advertising exposure with the consumption of energy drinks [29], and such exposure to energy-drink digital marketing has proven to be a major encouragement for young people to purchase and consume energy drinks [30]. About half of caffeine overdoses reported in the US involve adolescents, and more countries are now debating whether to restrict energy-drink sales and marketing to adolescents [31].

Children and adolescents have spent more time online and engaged in excessive gaming, particularly during the COVID-19 phenomenon [32]. Gaming disorder is now included in the *International Classification of Diseases* (11th ed.), while the recent worldwide prevalence of gaming addiction was 3.05% [33]. In Taiwan, a study found that the prevalence of Internet gaming disorder among adolescents was 3.1% [34]. Internet gaming disorder (IGD) has been defined as persistent and recurrent use of the Internet to engage in games leading to clinically significant impairment or distress, while the proposed diagnosis of IGD in the *Diagnostic and Statistical Manual of Mental Disorders* (DSM-5) requires experiencing at least five or more out of nine potential criteria (i.e., the need to spend increasing amounts of time engaged in games, experiencing unpleasant symptoms when gaming is taken away, continued excessive use of games despite knowledge of psychosocial problems, jeopardizing or losing a significant relationship, job, or education or career opportunity because of participation in games) over a period of one year [35]. Prior studies have associated energy-drink consumption with longer screen times [36] and longer times for video game use [37]. However, the relationship between excessive gaming, digital marketing exposure, and energy-drink consumption among adolescents has not been sufficiently studied.

Studies have associated energy-drink consumption among adolescents with alcohol use, substance use, risky behaviors, and negative school experiences [20,29,38]. Energy drinks could potentially act as gateway drugs to other substances [39], while the adverse health effects due to the consumption of energy drinks with alcohol are growing [40]. Consumption of caffeine and ethanol simultaneously has been associated with increased risk-taking behaviors in adolescents such as impaired driving and increases in the use of illicit substances [41]. Other studies have associated adolescents’ energy-drink consumption with polysubstance use and risky behaviors [1,42]. At least one study has indicated that middle-school students are more vulnerable to the negative effects of energy drinks relative to the use of illicit substances [43]. In addition, studies have shown that the prevalence rate of energy-drink consumption is higher among boys than it is among girls [29,36,38,44].

Consumption of energy drinks by children and adolescents is a growing public health concern [45]. During the COVID-19 pandemic period, studies indicated that adolescents spent more time online and had problems with excessive gaming [46,47]. In addition, the growth of digital marketing for beverages including energy drinks has grown substantially during the COVID-19 pandemic [22]. However, only a limited number of studies have investigated the relationships between excessive gaming, energy-drink digital-marketing exposure, online marketing literacy, and energy-drink consumption. Similar to other countries, selling energy drinks in Taiwan is as common as other drinks that carry no age restriction for purchase. Adolescents who are not yet fully developed are more vulnerable to the adverse effects of energy drinks. However, no study has yet examined the relationships between excessive gaming, energy-drink digital-marketing exposure, and energy-drink consumption among adolescents. In addition, adolescents’ excessive gaming, digital marketing exposure, and energy-drink consumption are increasing in Taiwan. Thus, in this study, we aimed to examine the relationships between excessive gaming, online energy-drink marketing exposure, energy-drink advertising effects, energy-drink marketing literacy, alcohol use, and energy-drink consumption among adolescents in Taiwan.

### Theoretical Background

Advertisements are the main source of information about energy drinks, while half of adolescents reportedly do not know that energy drinks contain caffeine [48]. Many adolescents may be unaware or misinformed about the potential health effects and nutritional content of energy drinks [49]. Prior studies have indicated that media literacy plays a crucial role in preventing adolescents’ use of sweetened beverages and energy drinks [50,51]. Media literacy has been defined as the ability to access, analyze, evaluate, and create messages across a variety of contexts [52]. Advertising literacy refers to consumers’ knowledge, skills, and ability to recognize and critically reflect the tactics, intentions, and strategies that are used in persuasive attempts in the context of advertising [53,54]. According to the Persuasion Knowledge Model, consumers’ ability to cope with persuasive attempts depends on the type of persuasion, the persuading agent, and topic knowledge. Persuasion knowledge in the form of advertising literacy enables consumers to recognize and critically evaluate the advertising source, the intended audience, and persuasive sales intent and tactics [54]. 

However, the measurement of persuasion knowledge varies across studies. A meta-analysis study showed that persuasion knowledge cannot eliminate persuasive effects in the marketplace, and few studies have examined the effect of particular sources (e.g., social media advertising), and consumers’ characteristics that might trigger different degrees of critical thinking [55]. Prior studies have argued that children are unlikely to use their advertising knowledge as a critical defense due to immature executive functioning. These studies have suggested that advertising literacy be extended to conceptual advertising literacy (i.e., understanding selling intent, and persuasive tactics) and attitudinal advertising literacy (i.e., skepticism toward advertising and disliking of advertising) [56,57].

As more adolescents are exposed to embedded advertising, they are also more susceptible to online sponsored content [58]. A study indicated that adolescents showed compassion toward influencers, instead of having a critical perspective [59]. The Persuasion Knowledge Scales of Sponsored Content was developed to measure consumers’ persuasion knowledge of sponsored content, while this scale consisted of two components, including conceptual understanding of the intent, source, tactics of advertising, and evaluative beliefs about advertising [58]. To address these gaps, the present study applied the Persuasion Knowledge Model [54], advertising literacy [56], and the Persuasion Knowledge Scales of Sponsored Content [58] to explore how online energy-drink marketing and digital-marketing literacy influence the rates of adolescents’ energy-drink consumption.

## 2. Materials and Methods

### 2.1. Participants

Data for this cross-sectional study were collected in 2020 from thirty schools in urban and rural areas in Taiwan. The study included twelve schools in New Taipei, four schools in Taoyuan, four schools in Miaoli, three schools in Changhua, four schools in Yilan, and three schools in Hualien. Thirty schools were selected via probability proportional to size. Seventh-grade students (aged 13 ± 1 years) were recruited for the study. This study was a paper–pencil survey delivered in classrooms. Students spent about 20–30 min completing the questionnaire, while trained interviewers answered students’ questions during the survey. A gift of stationery was provided for each of the participating students, and about 1% of questionnaires were rejected due to missing data or nonsensical responses. A total of 2613 participants (male, 1228; female, 1369; urban, 1527; and rural, 1070) were included in this study. The response rate was 96%. 

### 2.2. Materials

A self-administered questionnaire was developed. The experts’ professions included public health, media literacy, digital marketing, excessive gaming, and adolescent health, and experts reported having more than 10 years of experience. The questionnaires were delivered to students in their classrooms by trained interviewers. Students were assured that their information would be protected and remain anonymous. Approval was obtained from the Institutional Review Board at National Taiwan Normal University (201905HS068). 

### 2.3. Measures

#### 2.3.1. Energy-Drink Consumption

Energy-drink consumption was measured with one item. Students were asked “How often do you drink energy drinks (e.g., Red Bull)?” The responses were rated on a 5-point scale: never (scoring 1), ever (scoring 2), seldom (scoring 3), sometimes (scoring 4), and often (scoring 5). Higher scores indicated higher levels of energy-drink consumption.

#### 2.3.2. Online Energy-Drink Marketing Exposure

Online energy-drink marketing exposure was measured with one item. Students were asked “How often do you see energy-drink (e.g., Red Bull) advertising or marketing on the Internet?” The responses were rated on a 5-point scale: never (scoring 1), seldom (scoring 2), sometimes (scoring 3), often (scoring 4), and always (scoring 5). Higher scores indicated higher levels of online energy-drink marketing exposure. 

#### 2.3.3. Energy-Drink Advertising Effect

The effects of energy-drink advertising were assessed according to the responses to two items: “I would try energy drinks because of game placement marketing” and “I would like the energy-drink brand because the energy-drink industry sponsors a Facebook sports club event”. The responses were rated on a 4-point scale: strongly disagree (scoring 1), disagree (scoring 2), agree (scoring 3), and strongly agree (scoring 4). Higher scores indicated higher levels of efficacy for energy-drink advertising. The correlation coefficient between the two items of the energy-drink advertising effect was 0.76.

#### 2.3.4. Online Energy-Drink Marketing Literacy

Online energy-drink marketing literacy was measured with 5 items adapted from advertising literacy [56]. Online energy-drink literacy included cognitive and affective literacy. Digital-marketing cognitive literacy refers to the general awareness of online marketing information and to the ability to acquire knowledge, while digital-marketing affective literacy refers to an individual’s ability to criticize online marketing information. The energy-drink digital-marketing cognitive literacy included three questions, such as: “the live broadcaster of the game mentioned in the live broadcast that he relies on a certain brand of energy drinks to refresh himself, which are marketing techniques”. Two questions were used to measure energy-drink digital-marketing affective literacy. A sample situation was “Energy-drink products should not be placed in the game”. Responses were rated on a 4-point scale: strongly disagree (scoring 1), disagree (scoring 2), agree (scoring 3), and strongly agree (scoring 4). Higher scores indicated higher levels of online energy-drink marketing literacy. The factor loadings for the energy-drink digital-marketing cognitive literacy ranged from 0.53 to 0.74. The average variance extracted (AVE) was 0.42, while the composite reliability (CR) was 0.68. The Cronbach’s alpha of the energy-drink digital-marketing cognitive literacy was 0.67. In addition, the correlation coefficient between the two items of the energy-drink digital-marketing affective literacy was 0.38.

#### 2.3.5. Alcohol Use

Alcohol use was measured with one item. Students were asked “How often do you drink alcoholic drinks?” The responses were rated on a 5-point scale: never (scoring 1), ever (scoring 2), seldom (scoring 3), sometimes (scoring 4), and often (scoring 5). Higher scores indicated higher levels of alcohol use. 

#### 2.3.6. Excessive Gaming

Excessive gaming was measured using the Chinese version of the ten-item Internet Gaming Disorder Test (IGDT-10), which is recognized as having both good validity and reliability [34]. The instrument consists of ten questions and was developed based on the *DSM-5* nine-item criteria of IGD, including preoccupation, withdrawal, tolerance, loss of control, giving up other activities, continuation, deception, escape, and negative consequences [34]. All students answered the IGDT scale. A sample question was “When you were not playing, how often have you fantasized about gaming, thought of previous gaming sessions, and/or anticipated the next game”? Each item was rated on a 3-point scale: never (scoring 1), sometimes (scoring 2), and often (scoring 3). Higher scores revealed higher levels of excessive gaming. The factor loadings for the IGDT-10 ranged from 0.47 to 0.70. The AVE was 0.33, while the CR was 0.83. The Cronbach’s alpha of the IGDT-10 was 0.83. 

### 2.4. Statistical Analysis

Statistical analyses were performed using SAS version 9.4 for Windows (SAS Institute, Cary, NC, USA). The Student’s *t*-test statistic was used to identify gender differences in energy-drink consumption, exposure to energy drink marketing on the Internet, energy-drink advertising effect, online energy-drink marketing literacy, alcohol use, and excessive gaming. Multiple regression models were adjusted for gender and area and used to estimate factors related to adolescents’ energy-drink consumption, including excessive gaming, online energy-drink marketing exposure, energy-drink advertising effect, energy-drink digital-marketing affective literacy, and alcohol use. Variance inflation factors (VIF) tests were conducted to detect multicollinearity among explanatory variables. 

## 3. Results

### 3.1. Energy-Drink Consumption and Excessive Gaming 

Overall, 18% of adolescents reported having ever used energy drinks, while the rate of boys who had ever used energy drinks (22.6%) was higher than that for girls (12.8%). About 8% of adolescents reported having ever used alcohol. In addition, boys had higher levels of gaming addiction (Mean = 3.78) compared with levels for girls (Mean = 2.43) (Table 1). 

### 3.2. Adolescents’ Online Energy-Drink Marketing Exposure and Literacy

Overall, about 75% of adolescents reported that they had seen energy-drink advertisements on the Internet in the past year. By gender, boys reported higher levels of the effects of energy-drink advertising, which resulted in higher levels of energy-drink consumption. By contrast, girls reported higher levels of online energy-drink marketing cognitive literacy. In addition, boys reported higher levels of gaming addiction compared with the levels reported by girls (Table 1). 

### 3.3. Factors Related to Adolescent Consumption of Energy Drinks

Multiple regression analysis results indicated that gender, online energy-drink marketing exposure, energy-drink advertising effect, energy-drink digital-marketing affective literacy, alcohol use, and excessive gaming were significantly related to energy-drink consumption, while there was no multicollinearity among the explanatory variables (VIF < 1.2) (Table 2). Adolescents who were male, reported higher levels of online energy-drink marketing exposure, reported higher levels of the effects of energy-drink advertising, reported drinking alcohol, and reported excessive gaming were positively associated with energy-drink consumption. Adolescents who reported higher levels of online energy-drink marketing-affective literacy, however, were negatively associated with energy-drink consumption. 

## 4. Discussion

As far as we could ascertain, this is the first study to examine factors such as excessive gaming, online energy-drink marketing exposure, and energy-drink digital-marketing literacy, in order to gauge the association with energy-drink consumption among adolescents. Our results positively associated adolescent excessive gaming with energy-drink consumption. Studies have shown the prevalence of energy-drink marketing, which has the most exposure across social media and livestreaming platforms such as Twitch, Facebook gaming, and YouTube gaming [22,23,24]. Studies have also associated energy-drink consumption with video game use and health-risk behaviors and have suggested that programs designed to mitigate the effects should consider the clustering of energy-drink consumption with other unhealthy behaviors [36,60]. These studies have shown that excessive gaming might promote energy-drink consumption through online energy-drink marketing exposure, while energy-drink consumption might also aggravate excessive gaming behavior. Future studies are needed to explore the potential causality between excessive gaming and energy-drink consumption.

In the present study, three-fourths of adolescents reported seeing energy-drink marketing on the Internet in the past year, while exposure to online energy-drink marketing and the effects of advertising were associated with adolescent energy-drink consumption. The previously mentioned Canadian and Australian studies showed that energy-drink marketing is popular on digital platforms that target young people [24,27], and those studies positively associated energy-drink digital marketing with energy-drink consumption [24,27]. Another study positively associated adolescent energy-drink initiation with frequent energy-drink advertisement exposure [29]. To counter the effects of digital marketing on adolescent health, researchers have suggested regulating all forms of marketing, which includes Internet marketing [24]. 

In addition, the present study negatively associated online energy-drink marketing-affective literacy with energy-drink consumption, while online energy-drink marketing cognitive literacy was not associated with energy-drink consumption. Perhaps, adolescents spend more time online, and they might be more familiar with persuasion strategies particular to cognitive marketing literacy, but not to affective literacy. Discussion in a prior study suggested that advertising avoidance could be more positively related to evaluative literacy than to conceptual literacy [58]. Another study showed that different processes of persuasion were effective at different ages due to literacy levels [61]. A prior study showed that advertising literacy may reduce the impact of advertising unhealthy foods [62], while another study showed that individuals with higher sugar-sweetened beverage literacy consumed fewer sugary drinks [63]. A US study showed that energy-drink manufacturers primarily target youth with advertising. Those researchers suggested that adolescents’ energy-drink media literacy should be enhanced [64]. 

The present study positively associated adolescents’ energy-drink consumption with alcohol use. Longitudinal studies have also indicated that the use of energy drinks in early adolescence increases the later risk for alcohol use [65,66,67]. The US national survey positively associated energy-drink consumption with alcohol, cigarette, and illicit drug use [68], while the Canadian study showed that adolescents who used energy drinks were more likely to consume alcohol mixed with energy drinks [69]. A prior study has shown that adolescents who consume alcohol mixed with energy drinks have an increased risk of illicit drug use [70]. Schools should implement energy-drink-use prevention education to prevent further alcohol and substance abuse. 

In the present study, boys reported higher levels of energy-drink consumption and greater effects of energy-drink advertising. These results were consistent with prior studies [29,38,44] that showed boys used energy drinks more frequently than girls. Existing studies have shown different patterns of energy-drink consumption for boys and girls, which suggests that sex-tailored interventions could be relevant to reduce energy-drink consumption in adolescents [16,36].

## 5. Limitations

The present study had some limitations that should be considered when interpreting these results. First, this was a cross-sectional study and could not examine the causal relationships between energy-drink consumption, excessive gaming, and other related factors among adolescents. Second, reports by adolescents of online energy-drink marketing exposure and energy-drink consumption were collected via a self-reported questionnaire, which could lead to potential recall bias. Third, social-desirability bias could have had an influence on the truthfulness of the reports of excessive gaming and alcohol use. However, confidentiality was emphasized, and trained investigators collected the questionnaires. Fourth, this study only asked about the frequency of energy drink and alcohol drinking, without asking about the amount of drinking. Future studies could further collect the frequency and quantity of energy-drink and alcohol use to assess the severity of energy-drink and alcohol consumption. Fifth, the measurement of energy-drink digital cognitive and affective marketing literacy was assessed by asking only a few questions. Future studies could develop scenarios regarding online energy-drink marketing literacy for adolescents to improve the scale of validity and reliability. Finally, it is difficult to compare the results of studies conducted on different, incomparable populations and in different research periods. Despite these limitations, the strength of this study was a large sample size that was used to examine the relationships between adolescents’ excessive gaming, online energy-drink marketing exposure, advertising effect, marketing literacy, and energy-drink consumption.

## 6. Implications

This study positively associated online energy-drink marketing exposure with energy-drink consumption, while digital-marketing affective literacy was negatively associated with energy-drink consumption. Children and adolescents are vulnerable to digital marketing, and research suggests that schools could implement advertising literacy training for children and adolescents to cope with digital persuasion tactics and to empower parents to help their children more critically cope with the tactics of digital marketing [71]. Another study found that psychological reactance and sugar-sweetened beverage (SSB) media literacy played different roles in the sports and energy drink models and suggested the prevention messages in limiting energy drink consumption should be targeted and specific [51]. Another study showed that young people whose parents critiqued media messages reported more critical thinking skills, which predicted less interaction with alcohol brands on social media and fewer allusions towards the use of alcohol [72]. A study found that media-literacy-based, family-centered intervention could empower parents and improve youths’ critical thinking to reduce the negative effects of food marketing [73]. Future studies could develop parent–child co-learning programs focused on energy-drink conceptual and attitudinal digital-marketing literacy. 

In addition, this study associated excessive gaming and energy-drink marketing exposure with energy-drink marketing literacy. Prior studies have suggested educating adolescents to understand the dangers of excessive caffeine intake resulting from energy-drink consumption, and also suggest implementing policy interventions to restrict the sales of energy drinks and energy-drink marketing [25,41,45,74]. Another study also found that sugar-sweetened beverage (SSB) perceptions and media literacy were associated with SSB consumption and suggested education and policy interventions such as excise taxes and limits on marketing to youth [50]. *The Indian Academy of Pediatrics Guidelines* recommend that caffeinated energy drinks should not be consumed by children and adolescents and banning of screen/print/digital advertisements of energy drinks [75]. Schools should educate adolescents regarding the risks of energy-drink consumption and excessive gaming, while governments should regulate digital marketing to reduce risks to children and adolescents.

## 7. Conclusions

The present study positively associated online energy-drink marketing exposure and advertising with energy-drink consumption, while energy-drink digital-marketing affective literacy was negatively associated with energy-drink consumption. In addition, gaming addiction and alcohol use were positively associated with adolescents’ energy-drink consumption. Future studies could further examine potential causal relationships between gaming addiction, energy-drink marketing exposure, and energy-drink consumption. Governments could regulate the digital marketing of energy drinks and other unhealthy beverages to prevent negative health consequences, while schools could strengthen the particular effectiveness of students’ digital-marketing literacy and critical literacy skills in order to empower children and adolescents to resist the growth of the sponsored content and persuasive tactics used in the digital marketing of unhealthy beverages.

## Figures and Tables

**Table 1:** Gender differences in energy-drink consumption and related factors.

	Overall		Girls		Boys		*p* Value
	Mean	SD	Mean	SD	Mean	SD	
Online energy-drink marketing exposure	2.53	1.23	2.55	1.21	2.51	1.26	0.433
Energy-drink advertising effect	1.51	0.64	1.44	0.57	1.58	0.70	<0.001
Energy-drink digital marketing cognitive literacy	2.83	0.60	2.87	0.52	2.78	0.67	0.001
Energy-drink digital marketing affective literacy	2.78	0.67	2.80	0.61	2.77	0.73	0.281
Energy-drink consumption	1.55	1.03	1.41	0.88	1.71	1.14	<0.001
Alcohol use	1.28	0.70	1.35	0.77	1.21	0.60	<0.001
Gaming addiction	3.06	2.52	2.43	2.36	3.78	2.50	<0.001

N = 2613. *T*-tests conducted.

**Table 2:** Factors related to energy-drink consumption.

	Β	SE	*p*	VIF
Gender (Female = 0, Male = 1)	0.24	0.04	<0.001	1.11
Area (Urban = 0, Rural = 1)	0.02	0.04	0.563	1.04
Online energy-drink marketing exposure	0.09	0.02	<0.001	1.07
Energy-drink advertising effect	0.51	0.03	<0.001	1.13
Energy-drink digital marketing cognitive literacy	0.01	0.03	0.696	1.19
Energy-drink digital marketing affective literacy	−0.06	0.03	0.033	1.18
Alcohol use	0.27	0.03	<0.001	1.04
Excessive gaming	0.02	0.01	0.002	1.14

N = 2613. Multiple regression conducted.

## Data Availability

The data presented in this study can be requested and provided.
